# Effects of Sheltering on Behavior and Fecal Corticosterone Level of Elderly Dogs

**DOI:** 10.3389/fvets.2016.00103

**Published:** 2016-11-21

**Authors:** Katsuji Uetake, Chu Han Yang, Aki Endo, Toshio Tanaka

**Affiliations:** ^1^Laboratory of Animal Behavior and Management, School of Veterinary Medicine, Azabu University, Sagamihara, Japan; ^2^Kanagawa Animal Protection Center, Hiratsuka, Japan

**Keywords:** animal shelter, animal welfare, behavioral change, fecal corticosterone, stereotypic behavior, stress response, elderly dogs

## Abstract

In Japan, the human population is aging rapidly, and the abandonment of dogs by the elderly people who have died or been hospitalized becomes a problem. It is hypothesized that elderly dogs have difficulty adapting to the novel circumstances when brought to an animal shelter. Therefore, the objective of this study was to assess stress levels and demonstrate stress responses of elderly dogs just after admission to an animal shelter. As stress indicators, fecal corticosterone levels and changes in the ethogram of the dogs were investigated during the first week of admittance. Fecal corticosterone levels (mean ± SE) stayed high during the first week of residence, although they fell gently from the day after admittance (16650.1 ± 3769.7 ng/g) to the seventh day (12178.4 ± 2524.4 ng/g) (*P* < 0.001). The proportions of behavioral expressions changed as the days passed (*P* < 0.001). In particular, stereotypies decreased from 35.7% on the first day to 2.6% on the sixth day, and time spent sleeping increased from 0.0 to 42.7%. These results indicate that elderly dogs admitted to an animal shelter seem to behaviorally adapt themselves to their novel circumstances but might be stressed even on the seventh day of residence.

## Introduction

A central problem in animal welfare of pet dogs is abandonment by their owners. Abandoned dogs are commonly brought to animal shelters, and they suffer many kinds of psychogenic stressors there (1). In particular, the stress level of dogs as assessed by plasma cortisol concentration was reported to be highest during the first 3 days in the shelter (2). As physiological indicators of stress, hypothalamic–pituitary–adrenal measures using blood, saliva, urinary, fecal, and hair glucocorticoids, and their metabolites are also common, as reviewed by Hennessy (1).

In addition to physiological stress responses, behavioral changes indicate that stress was observed when dogs were kenneled at the shelter. As behavioral indicators that give signs of stress in kenneled dogs, changes in activity levels as well as the expression of stereotypic behaviors and fear that dogs display at the shelter have been reported, as reviewed by Protopopova (3).

In Japan, the human population is aging rapidly, and the abandonment of dogs by the elderly persons who have died or been hospitalized has become one of the main reasons. These dogs are often old, too. It is hypothesized that the elderly dogs have difficulty in adapting themselves to the novel circumstances in the shelter. For example, Mongillo et al. (4) reported that aged dogs (7 years or older) cope less efficiently with emotional distress caused by a change of caretaker.

Therefore, the objective of this study was to assess the stress level and demonstrate stress responses of elderly dogs that were admitted to an animal shelter. As stress indicators, fecal corticosterone level and changes in their ethograms were investigated on a daily basis, especially focusing on the first week of admittance. Fecal corticosterone monitoring was applied because of its non-invasiveness to subject animals (5).

## Animals, Materials, and Methods

### Subjects and Housing

The 14 elderly dogs (8 years or older, range 8–13) admitted to the Kanagawa Animal Protection Center from April 2014 to September 2015 were candidates in this study. Of the 14 dogs that we started observing, 12 dogs (mean ± SD = 9.8 ± 1.9 years) completed the 7 days of data collection. Thus, subject animals totaled finally 12 dogs. There were 10 males and 2 females (all intact), and 6 mongrels and 6 purebred dogs. Pure breeds included Papillon (*n* = 1), Toy Poodle (*n* = 1), Miniature Dachshund (*n* = 2), English Cocker Spaniel (*n* = 1), and Border Collie (*n* = 1).

Subject dogs were caged individually in a room of a concrete building. Six small cages (0.8 m × 1.5 m × 0.7 m/cage) and eight large cages (1.0 m × 2.0 m × 2.5 m/cage) were adjacently installed in the room. Both size cages were bare and high-floored. Each dog was allocated to a cage depending on its body size. In this study, four mongrels, English Cocker Spaniel, and Border Collie were kept in large cages. Dogs were kept indoors all day without outdoor exercise or positive human contact such as a walk. All dogs were fed commercial dog food twice a day (0900 and 1700 hours) and allowed free access to water. Room temperature was controlled at around 25°C using an air conditioner. Room lights turned on at 0730 hours and off at 1700 hours every day. The illuminance level of the room at the time of the lighting was approximately 40 lx. The noise level was not measured.

### Fecal Sampling and Enzyme Immunoassay

There is a report that canine fecal corticosterone levels increase during the first 24 h after ACTH administration and have returned to baseline by 48 h postinjection (5). Considering this time lag, feces were collected from individuals daily from the day after the admittance till the seventh day during the morning routine. Fecal samples were stored in polypropylene bags and immediately frozen at −80°C until drying.

The fecal samples were dried in an oven (SDN27; Sansyo, Tokyo, Japan) at 100°C for more than 16 h and then powdered using a mill (IFM-800DG; Iwatani, Osaka, Japan). Powdered feces were extracted using a vortexing method modified from Wasser et al (6). Briefly, 0.1 g of dried sample was placed in a 2 ml microtube with 1 ml of 95.5% ethanol, capped, vortexed (5 min) using a vortex mixer (Vortex-Genie2; M&S Instruments Inc., Osaka, Japan), and then centrifuged (CN-820; AS ONE Corp., Osaka, Japan) for 5 min at 1050 × *g*. The fecal corticosterone concentration of the supernatant was determined using an enzyme immunoassay kit (ADV900-097; Cosmo Bio Co. Ltd., Tokyo, Japan). The extraction efficiency of exogenous corticosterone using this method was 65.1%. Intra-assay coefficients of variation were <10% for all assays. All fecal concentration data are expressed as nanograms per gram of dry feces.

### Behavioral Observations

Behavioral observations were conducted from 1400 to 1600 hours on the first 6 days of residence. Behavioral activities of each dog were videotaped using a digital camcorder (Handycam HDR-PJ210; Sony, Tokyo, Japan) mounted on a tripod. Table 1 specifies the behaviors recorded in this study. As a recording method, instantaneous scan sampling at 1-min intervals was adopted.

**Table 1:** **Ethogram used for behavior observations**.

Behavior	Description
Ingesting	Eating food or drinking water
Resting	Standing or lying without any activity. Eyes are open
Sleeping	Lying with eyes closed
Grooming	Licking or slightly biting the limbs or body
Exploring	Sniffing the cage bars or floor
Stereotypies	Repetitive circling or pacing back and forth
Other	Urination, defecation, and others

### Statistical Analyses

Data were analyzed using the statistical software program Statcel4 (OMS Publishing, Tokyo, Japan). In normality test, data on fecal corticosterone level did not follow normal distribution (all sampling days: χ^2^ ≥ 4.26, df = 1, *P* < 0.05). Thus, differences in fecal corticosterone level between days in shelter were tested with Friedman repeated measures analysis of variance on ranks, and *post hoc* Steel–Dwass multiple comparison. To compare the proportion of behavioral expressions between days in shelter, the relationship between the sample points of seven behavioral activities marked within a recording session (2 h/day) and day in shelter (the first to sixth day of residence) was determined using contingency-table analysis. The protocol was reviewed and approved by the Azabu University Research Service Division (2014K15).

## Results and Discussion

**Figure 1:**
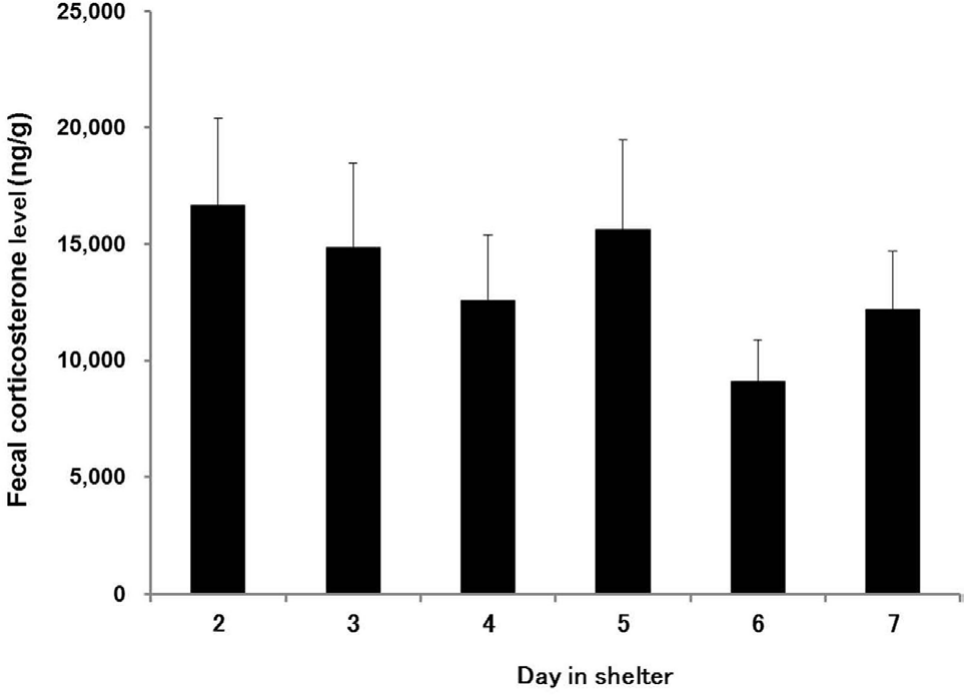
**Mean fecal corticosterone levels of elderly dogs sampled from the day after admittance to the seventh day**. Vertical lines represent SEMs. The effect of day in shelter on fecal corticosterone level was significant (χ^2^ = 23.19, df = 5, *P* < 0.001).

The effect of day in shelter on fecal corticosterone level was significant (χ^2^ = 23.19, df = 5, *P* < 0.001). Fecal corticosterone levels gently fell from the day after admittance (mean ± SE: 16650.1 ± 3769.7 ng/g) to the seventh day (12178.4 ± 2524.4 ng/g; Figure 1). However, there was no significantly different combination of days in the shelter in *post hoc* multiple comparisons.

**Figure 2:**
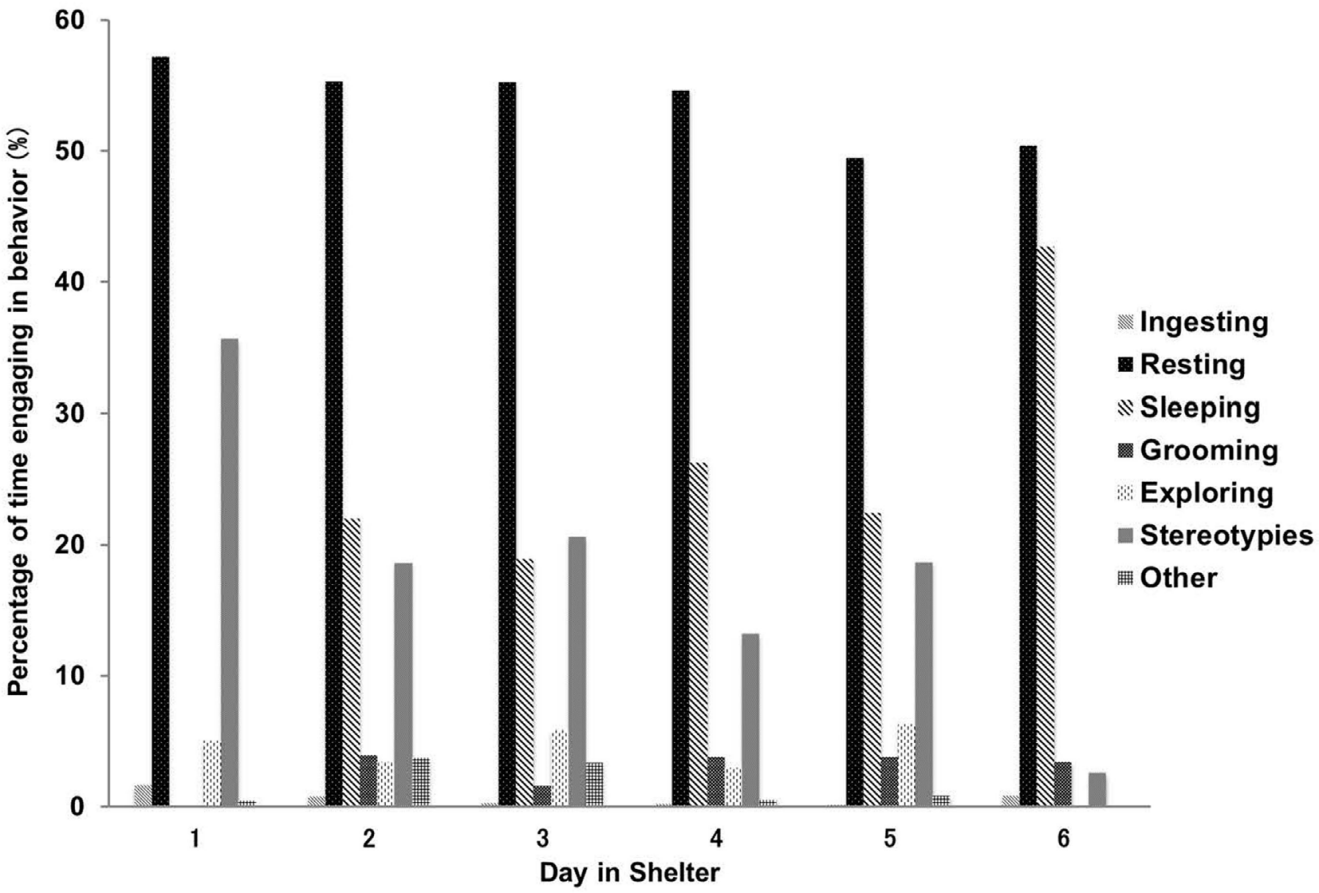
**Mean proportions of behavioral expressions of elderly dogs from the first to sixth day of residence**. The proportion of behavioral expression was significantly different between days in shelter (χ^2^ = 120.36, df = 30, *P* < 0.001).

The proportion of behavioral expressions was significantly different between days in shelter (χ^2^ = 120.36, df = 30, *P* < 0.001). Behavioral activities that showed a big change during the first 6 days of admittance were stereotypies and sleeping behavior. The former gradually decreased from 35.7% on the first day to 2.6% on the sixth day, and the latter increased from 0.0 to 42.7% (Figure 2).

As far as we saw a change in behavioral expression of the elderly dogs, they seem to habituate themselves to the novel circumstances of an animal shelter in the first week of residence. A decrease in the expression of stereotypies or repetitive behavior is a good indication of environmental adaptation. In this study, behavioral observations were conducted in a time zone (1400–1600 hours), where caretakers did not exist nearby. Thus, increase in sleeping behavior in this time would be a good indication, too.

On the other hand, their fecal corticosterone levels were still high even on the seventh day of residence (12178.4 ± 2524.4 ng/g). It was more than 10-fold the mean baseline level of dogs that had regular contact with humans at the same shelter (e.g., dogs used for dog training classes: 1035.9 ± 179.9 ng/g) (7). It is reported that the stress level of dogs admitted to a shelter is the highest in the first 3 days in the shelter, with a gradual waning thereafter (2). We cannot simply compare stress levels across studies because besides the age of the dogs many factors differed (e.g., measurement in the plasma vs. in the feces, different shelters, different dog breeds, etc.). However, when we synthesize these behavioral and physiological indicators, it seems that the elderly dogs need more than 1 week to habituate themselves adequately to the novel circumstances in the shelter.

As a practical way of reducing the stress of dogs in shelters, it is recommended that shelter staff members spend time interacting with the resident dogs (8). However, Mongillo et al. (4) have reported that aged dogs cope less efficiently with the emotional distress caused by a change of caretaker. For this reason, they need a longer time to develop trust for unfamiliar staff members and relax their guard. Gaultier et al. (9) reported that an alternative way to reduce stress linked to social separation is using a dog-appeasing pheromone.

In conclusion, the elderly dogs newly admitted to an animal shelter seem to behaviorally adapt themselves to the novel circumstances but might be stressed even on the seventh day of residence. Further studies are needed to clarify how long elderly or aged dogs need to overcome the stress caused by abandonment by their owners and to treat it adequately.

## Author Contributions

KU, CY, and AE designed the study through discussion, and TT gave advice about the experimental design. CY and AE performed the data collection, and KU and CY performed statistical analyses. All the authors interpreted data. KU wrote the manuscript, and CY, AE, and TT contributed to the manuscript. All the authors approved the final version of the manuscript.

## Conflict of Interest Statement

The authors declare that the research was conducted in the absence of any commercial or financial relationships that could be construed as a potential conflict of interest.
